# The Epistemological Foundations of Freud’s Energetics Model

**DOI:** 10.3389/fpsyg.2018.01861

**Published:** 2018-10-11

**Authors:** Jessica Tran The, Pierre Magistretti, François Ansermet

**Affiliations:** ^1^Agalma Foundation, Geneva, Switzerland; ^2^Départment of Psychoanalytical Studies, Paris 7 Diderot University, Paris, France; ^3^Institute of Medical Humanities, Université de Lausanne, Lausanne, Switzerland; ^4^Department of Psychiatry, Faculty of Medicine, Brain Mind Institute, Swiss Federal Institute of Technology in Lausanne, Lausanne, Switzerland; ^5^Division of Biological and Environmental Science and Engineering, King Abdullah University of Science and Technology, Thuwal, Saudi Arabia; ^6^Department of Psychiatry, Faculty of Medicine, University of Geneva, Geneva, Switzerland

**Keywords:** energy, Freud, thermodynamics, epistemology, Helmholtz, physics

## Abstract

This article aims to clarify the epistemological foundations of the Freudian energetics model, starting with a historical review of the 19th century scientific context in which Freud’s research lay down its roots. Beyond the physiological and anatomical references of *Project for a Scientific Psychology* (Freud, 1895a), the physiology Freud makes reference to is in reality primarily anchored in an epistemological model derived from physics. Whilst across the Rhine, the autonomy of physiology in relation to physics was far from being accomplished, as a counterpoint, in France, the revolution in physiology driven by Claude Bernard established itself autonomously from physics,. In contrast, Freud’s scientific landscape is entirely dominated by the physics elevated to the rank of an ideal science. The influence of Helmholtz, who is both a medical doctor and a physicist, has a determining influence on Freud’s training. The discoveries in physics at that time, in particular the formulation of the principle of ‘conservation of force’ – first principle of thermodynamics – will constitute the points of reference upon which Freud will elaborate his energetics model, then subsequently, the idea of economy in his metapsychology. In this way we can trace both the historic and epistemological path that led Freud from a concept based on physics, and more specifically thermodynamic energy, to an idea of nervous energy that constitutes the basis of the concept of “quantity” as it is stated as ‘first fundamental idea’ in *Project for a Scientific Psychology* (Freud, 1895a). This notion will subsequently evolve, and lead Freud to the introduction of the concept of ‘psychical energy,’ this time in a purely metapsychological sense.

## Introduction

The economics point of view of Freudian metapsychology today offers astonishing points of convergence with recent discoveries in contemporary neurosciences, in that it is based explicitly from its first formulations on an energetics model. In 1895 Freud had, in *Project for a Scientific Psychology*, proposed a first principle for the functioning of the psyche, the “principle of neuronal inertia” (Freud, 1895a, p. 296). Freud defined this as the tendency of neurones to divest themselves completely of the quantities of excitation (endogenous or exogenous) that erupt into the psychical apparatus. The primary function of this apparatus would therefore be to reduce to the lowest level possible – ideally a ‘level = zero’ - the quantity of free energy. This fundamental hypothesis of all the economic dimension of Freudian metapsychology – which would be later refined in the definitions of the principle of pleasure and the death drive – has recently been put into perspective through the neuroscientific work of Karl Friston and his colleagues. Indeed Friston and his colleagues see cerebral functioning through a Bayesian approach, its aim being to avoid too great a variation in the quantity of free energy coming from our sensorial perceptions (both internal and external) on the basis of prediction of sensorial data. This offers an unexpected point of dialog between psychoanalysis and neuroscience centered on an energetics concept of cerebral function.

While the energetics concept of the psychical function proposed by Freud can offer fruitful points of discussion with neurobiology, it is important to return to the epistemological roots of Freudian energetics in order to pin-point their theoretical origins. The first arguments of *Project for a Scientific Psychology* (Freud, 1895a) do indeed seem to correspond to a biological, even neurobiological, model. In his text, Freud introduces a ‘theory of neurons’. This theory constitutes one of the ‘two fundamental concepts’ that he bases his work on, alongside the concept of ‘quantities’ understood in terms of energy. However, a brief excursion into the history of the epistemological origins of his concept of nervous energy will enable us to glimpse that this is not truly a biological model. It is on the contrary a paradigm that is radically linked to physics, inspired by work done on the conservation of force, and profoundly influenced by the School of Helmholtz. This journey, following the origins in physicalism of Freudian energetics, will thus serve as a basis for a model dialog between psychoanalysis and neurosciences, where the heterogeneous epistemological roots of these two disciplines can be taken into account.

## The Physicalist Paradigm of the Berliner Physikalische Gesellschaft

In France, beginning in the 1860s, the “revolution in physiology” (Prochiantz, 1990) brought about by Claude Bernard made a radical epistemological leap by establishing physiology as a discipline in itself. This was autonomous in relation to physics–chemistry (even though Claude Bernard will always postulate a strict physico-chemical determinism of the vital phenomena). Bernard did indeed claim the existence of an undeniable singularity of the *vital aspect* amongst all other physico-chemical aspects, while at the same time strongly criticizing any vitalist stance. According to him, within the organic, “the mechanism is special […], the agent is specific, though the result is identical. No single chemical phenomenon occurs within the body similarly to outside of it” (Bernard, 1885, p. 219). It was in this singularity of the vital mechanism that the concept of homeostasis was rooted, and was later theorized by Cannon, to then be elevated to the rank of physiological mechanism central to all biology.

It was at a time exactly contemporary to the last Bernardian conceptualizations (the *Leçon sur les phénomènes de la vie* are published in 1878, the year Claude Bernard dies), but in a radically different geographic and scientific context, that Freud undertook his medical studies at the Vienna Faculty in the autumn of 1873 (Jones, 1953). Across the Rhine the autonomy of physiology in respect to physics was far from having been realized, and it was on the contrary within an epistemological paradigm that was decidedly antagonistic to that of French biology, that Freud’s scientific training was conducted. At the conclusion of his third year Freud joined Ernest Brücke, whom he saw as a “model” (Freud, 1925d, p. 9), at his laboratory of physiology. Besides the respect and admiration Freud felt for this undisputed master (Jones, 1953), this filiation bore witness to an affiliation to a whole scientific paradigm of which Freud will make himself the heir. As Jones underlines, Brücke’s institute was closely connected with the school of Helmholtz. The story of this scientific movement had begun in the 1840s with the friendships between different physiologists trained in Johannes Müller’s theories on the energy specific to nerves (Assoun, 1981). Du Bois-Reymond, Brücke, Helmholtz, and Ludwig came across as medical doctors imbued with a real “crusading spirit” (Jones, 1953) who, as Du Bois-Reymond reported, had “pledged a solemn oath to put into effect this truth: “No other forces than the common physical-chemical ones are active within the organism.”” (Jones, 1953, p. 40). Although they all had medical training, their scientific ideas where totally subordinated to physics. This small group, increased by the addition of new members, young student physicists and physiologists in leagued against vitalism, became in 1845 the *Berliner Physikalische Gesellschaft*, Berlin Physical Society (Jones, 1953). Within less than 30 years they will dominate the German scientific landscape becoming the most influential professors of medicine and physiology of their time, and in turn training a whole generation of students to which Freud and Wundt belonged. The majority of these professors can be equated with what Paul-Laurent Assoun calls the figure of the “doctor-physician,” of whom Fechner, Helmholtz, or Lotze will be the principle representatives: “all of them come to physics through medicine or via physiology” (Assoun, 1981, p. 59). For some of them, psychology would constitute the final stage of the journey. It is this scientific practice characterized by its diversity and lack of specialization that Freud would inherit during his years of training at the Brücke Institute. However, it is physics that constitutes for all the related disciplines the epistemological model *par excellence*. It can be observed that the German school of physiology positioned itself in a movement that was the exact opposite of Bernardian physiology: where in France there was a call for a certain independence of physiology as a separate science, autonomous from physics, the Berlin medical practitioners would on the contrary seek to subordinate physiology to physics, making it an extension of the latter. Brücke thus appears as one of the paradigmatic representatives of this trend:

“What is physiology in Brücke’s eyes? This is not a pointless question to ask, for the mistake would be to project onto that word the concept formed in the parallel tradition, in France, by Claude Bernard. Physiology for Brücke, leader of the Berlin Physics Society around 1845, is an extension of physics. It has as its object specific physico-Chemical systems, the organisms […]. The physiologist is none other than the physicist of organisms” (Assoun, 1981, pp. 101–102).

## The Influence of Helmholtz

Thus it was to a physiology radically subordinated to physics, the overruling dominant science – to which all natural phenomena must be brought back, including those relative to living organisms – that Freud would make himself heir. It is within this orientation that he trained at the Brücke Institute. However the dominant influence of Helmholtz, who of all the scientists at the Berlin Physics Society was without doubt the most eminent, needs to be emphasized. Freud considered him one of his “idols” (Jones, 1953), and would always regret not having had the opportunity to meet him in person. Helmholtz, perfect embodiment of the figure of the ‘doctor-physician,’ would dominate the German university scene at a time when it was becoming a model and a center of European science. According to him: “all natural phenomena must be brought back to the movement of material particles endowed with invariant driving forces, dependent only on their spatial location” (Prigogine and Stengers, 1979, p. 148). In this he made himself the advocate of an understanding of nature based on mechanical ideas, and the majority of the physiologist of the powerful German school (Liebig, Ludwig, Müller, Du Bois-Reymond, Virchow, Brücke) would adopt his concept according to which “the physico-chemical functioning of the living organism is subject to the same laws as inanimate matter, and must be studies within the same terms” (Prigogine and Stengers, 1979, p. 148).

To understand the influence of this physicalist model in 19th century Germany, it is important to underline that it made its appearance as a reaction to the influence of Schelling’s *Naturphilosophie.* A Romantic philosophy that argued for a pantheist monism close to mysticism (Jones, 1953). This philosophy saw nature as a unique fundamental great organism, unified by general laws, by a single principle of causality, and without remainder (Prigogine and Stengers, 1979). While this idealistic concept of nature was spread across all of Europe, German *Naturphilosophie* was characterized by its aspiration to what Schelling described as a ‘speculative physics.’ It is against this romantic view of a speculative philosophy, for which Helmholtz or Du Bois-Reymond feel a real aversion, that the physicalism of the Berlin Physics Society positioned itself (Meulders, 2001). Freud had been tempted in his youth by these ideas before definitively converting to the views of physicalist science. It was, according to Ernest Jones, under the influence of Goethe that Freud went through a brief period of *Naturphilosophie*, before becoming enthused by the competing physical physiology. In *The Interpretation of Dreams* (Freud, 1900a), Freud mentions that 1 day a violent philosophical discussion with a student, partisan of natural philosophy, nearly led him to a duel. Jones comments on this reactive movement in these terms:

“Physical physiology – although not by itself – overthrew this philosophy and took its place. As has happened before, the conqueror introjected the emotionalism of the victim. ‘Unity of science,’ ‘science,’ ‘physical forces’ were not merely directing ideas or hypotheses of scientific endeavor: they became almost objects of worship. They were more than methods of research – they became a *Weltanschauung.*” (Jones, 1953, p.43)

This very strong physicalist scientific ideal, almost raised to a status of religious conviction, appears then to be a virulent reaction to any vitalist views. The radical character of this epistemological model, where philosophy finds itself completely subjugated to physical science, contrasts in a notable way with the Bernardian view which according to Canguilhem constitutes a third pathway between vitalism and reductionism (Canguilhem, 1994). Thus, as Alain Prochiantz points out, up until Claude Bernard “the relationship of biology to physics was divided between complete assimilation in a physicalist reductionism, and radical separation within French vitalism or the German natural philosophy” (Prochiantz, 1990, p. 35). Bernardians therefore rejected both mechanism and vitalism to constitute a third position, adjusting the technique of biological experimentation to the singularity of its object of study, the living.

If the radical physicalism of the German school of physiology is to be understood through the prism of this opposition to the dominant position of natural philosophy (a position whose influence on scientific speculation had been much stronger across the Rhine) it is also to be situated in the context of the discovery in physics of the conservation of energy. Helmholtz was one of the first theoreticians of the conservation of energy. We can, along with Prigogine and Stengers, note the paradoxical fact that the philosophical past of Germany had imbued the scientists, in spite of them, with “an idea far removed from the strictly positivist knowledge that they professed to practice: the idea that nature, in its entirety and without remainder, is unified by a general law, by a single principle of causality” (Prigogine and Stengers, 1979, p. 175). Something akin to a return of the suppressed *Naturphilosophie* against which they had positioned themselves.

## The Principle of the Conservation of Force

Unifying of physiology and physics resulted from this supposed universal principle of the conservation of energy, according to which “the sum of the forces remains constant in all isolated systems” (Assoun, 1981, p. 102). If Helmholtz was one of the first theoreticians of the principle of the conservation of force, it is Mayer who is considered to have introduced the fundamental distinction between force and matter. Thus it is not surprising, in view of the continuity that existed between physiology and physics to which it is subordinated, that one of the major discoveries of 19th century physics, the principle of the conservation of energy introduced by Mayer in 1852, would have significant consequences for the development of physiology, psychology, and ultimately psychoanalysis (Assoun, 1981). However it is probable that for these two figures of the ‘doctor-physicist’ that are Hemholtz and Mayer – whose mixed practice was correlative of the porous nature of the boundaries between connected disciplines in the German scientific context – it was originally the studies of living organisms that conferred upon them the intuition of this principle. Their experimental and theoretical scientific practice was in reality so interconnected, moving between the study of living organisms and the inanimate, that it is difficult to establish the physical or physiological pre-eminence of the observation of the conservation of force (which will subsequently be reformulated as the principle of the conservation of energy). It would be more relevant, rather than to look for such a pre-eminence, to underline that these ‘great men’ of the 19th century [as Ostwald calls them in his biographical study (Ostwald, 1912)] were accustomed to a mental gymnastics that allowed them to move without difficulty from physiology to physics, and back again. Their experimental practice in one of these two sciences, leading them naturally to theoretical formulations that were equally valid in the other. This practice was justified by a presupposition inherited from Kantian philosophy: nature is ruled by the law of causality, in so far as all change in nature is due to a sufficient cause. As we have seen though, some remanent of the influence of the *Naturphilosophie* (to which they were in fact vigorously opposed) led these doctor-physicists to seek a single principle of causality that would unify nature (both organic and inanimate) in a whole without remainder (Prigogine and Stengers, 1979).

According to Prigogine and Stenger when Mayer, as a young doctor in the Dutch colonies of Java, observed the bright-red color of one of his patients’ blood, he concluded from this that since it was warmer in the tropics the inhabitants would need to burn less oxygen. On the basis of this observation, he made an assessment of the consumption of oxygen, which could be considered as a source of energy, and consumptions linked to the maintaining of body temperature with respect to thermic loss, and to manual labor. Mayer generalized the implications of this assessment (which already amounted to an interpretation with respect to the observed facts) to conclude the existence of a “single and indestructible force that is the basis of all phenomena both of living and inanimate nature” (Prigogine and Stengers, 1979, p. 175–176). This thesis of a single energetic principle offered to physiology the grounds for its claim to reduce the ‘vital process’ down to a mechanical chain of event (Assoun, 1981). Mayer’s observations in the tropics thus led him to argue that the body’s heat was the result of the chemical energy of food, and he went so far as to assert that the mechanical energy of muscles came from the same origin: mechanical energy, chemical energy, and heat would thus be equivalent and mutually convertible. Back in Germany, he established himself as a doctor and continued his research. He went on to demonstrate that there exists an equivalence between thermic and mechanical work, and calculated that the quantity of heat was equal to a given quantity of mechanical energy. He would present this thesis in 1842 in his *Remarks on the Forces of Inorganic Nature* (Brossollet, 2018).

As Paul-Laurent Assoun points out, “Mayer appears as the Lavoisier of the 19th century, perpetuating the grand principle of the conservation of matter” (Assoun, 1981, p. 60) turning it into a principle of conservation of force; which would become, after the introduction of the terms by Thomson in 1850, the principle of conservation of energy. This discovery could therefore, have come from physiology, to then be theorized and formulated in mathematical equations in the domain of physics, before returning to physiology where its consequences would lead to the development by Wundt of scientific psychology, on the basis of this same principle. Wundt would, it appears, have extended “for the first time, the law of conservation of force to the area of psychology” (Assoun, 1981, p. 60). The discovery of the principle of conservation of energy would also have a notable influence on the thinking of the young Freud. However, this circular phenomenon within German scientific knowledge makes it difficult to establish with certainty the provenance of this principle, whether it was from physiology or physics. Researchers like Mayer or Helmholtz moved into a larger unifying paradigm, where the absence of clear cut lines between disciplines, and a mixed scientific practice (combining both medical practice and research in physics) was completely foreign to the establishing of distinctions between disciplines. This was made possible in France by the advent, with Claude Bernard, of experimental physiology. We can thus argue, as Paul-Laurent Assoun proposes, that from the 1840s “a kind of practice is put in place, that comes simultaneously from physiology, physics, and chemistry; emerging from common and converging interests within a matrix of energetics” (Assoun, 1981, p. 60).

For Mayer then, the *vital aspect* resulted from the transformation of force or matter, and the task of physiology would consist henceforth in the investigation into the mechanisms of this transformation (Assoun, 1981). His work would also be in close relation with the experimental chemistry ushered into Germany by Liebig. Liebig who would contribute to the development of organic chemistry through the study of the chemical processes of living matter (it is indeed in Liebig’s review that Mayer’s historic memoir on the conservation of force was published). The chemistry of Liebig is essentially analytic: his method consisted in an analysis of the constituent parts of organisms, and he held that it was possible to go from a vegetable compound to an animal compound through the subtraction of constituents (Assoun, 1981). As Paul-Laurent Assoun remarks, this analytic organic chemistry of Liebig’s, in close relation with the work of Mayer, would make a deep impression on Freud who, in giving the name of ‘psychoanalysis’ to his discovery, would borrow specifically the term ‘analysis’ from the breaking down of the chemical compounds in experimental chemistry inspired by Liebig (Assoun, 1981).

In 1842, in his *Remarks on the Forces of Inorganic Nature*, Mayer did not yet refer to the concept of energy (which will only appear after 1850 in the writings of Thomson, then Rankine), but held to the definition of the concept of ‘force.’ Thus “the Mayerian project was clearly to ensure the epistemological promotion of the idea of ‘force”’ (Assoun, 1981, p. 159). While in Germany, the dynamical view inherited from Leibniz and Kant had made force the primary concept, the school of Laplace considered it as an emanation derived from matter. Mayer did not adopt a strict dynamism, since he established an analogy between matter and force. According to him, matter was the fundamental concept of chemistry, ponderable, transformable, and quantitatively indestructible during chemical reactions (where mass would always be conserved, although the quality, for example of oxygen and hydrogen, will not be seen in water) (Locqueneux, 2009). We can already observe here, in chemistry, the hypothesis of a qualitative transformation that nevertheless implies that quantity remains the same; something that Mayer would also apply to force. As Robert Locqueneux underlines, “the role held by matter in chemistry should, according to Mayer, be played by force in physics” (Locqueneux, 2009, p. 113). Force would therefore also be an indestructible and transformable entity just like matter; but, unlike matter, imponderable. Thus, the inanimate forces of nature could take on multiple qualitative forms: kinetic, thermic, magnetic, electrical, or chemical force. These are qualitative forms that would be phenomenologically distinct manifestation of a same entity, an *Urkraft*, ‘elemental force,’ (a quest for a primal unique force that is not dissimilar to the *Naturphilosopie’s* project, although Mayer was opposed to this). Yet, Mayer was the first to try to establish a quantitative science of force and no longer an only qualitative one: he determined through calculation the mechanical equivalent of heat. The aim was not here to make heat a kind of movement, but to “determine the equivalence between the disappearance of a quantity of heat, and the simultaneous production of movement” (Locqueneux, 2009, p. 114). In this way he would propose an equation for the equivalence between heat and movement (that is to say work in the mechanical meaning of the word). For indeed, according to Mayer,

“If two metals are rubbed together, there is movement that disappears and heat that develops; hence the question: if the movement is the cause of the heat […]. If we cannot account for the disappearance of the movement, without admitting a causal relation between the movement and the heat; it is not possible to understand, without admitting this connection, how the heat develops. It is demonstrated that, in many cases, the disappearance of the movement has no other appreciable consequences than this production of heat” (Mayer, In: Locqueneux, 2009, p. 131).

Mayer then, assumed that the latent heat was transformed in its entirely into a quantity of work: this was a qualitative transformation (the mechanical force was turned into a force of a different nature on a phenomenal level, heat), but one that implied a conservation, an equivalence, from a quantitative point of view. He illustrated this with the example of hydraulic mechanisms where the movement, as it destroys itself, provokes a considerable quantity of heat; and also with steam machines where the reverse takes place, it is the heat that provokes movement. There is then a principle of equivalence, that also implies a reversibility, between movement and heat, as two qualitatively distinct aspects of a same original force (Locqueneux, 2009).

Three years after this work on the forces in inanimate nature, Mayer would return to questions of physiology and write a memoir on *The Motions of Organisms and their Relation to Metabolism* (1845). He described his project as a desire to “fill the chasm that separates exact physics and physiology” for which “a method that would seek to bring together these two sciences under this one perspective, would be invaluable for physiology” (Mayer, 1845, In: Assoun, 1981, p. 163). This union between physiology and physics was thus sealed by the reunion of the heterogeneous phenomena observed by these two disciplines under an overruling principle. This overruling principle would be the conservation of force – subsequently translated into conservation of energy. In both organic and inorganic phenomena, there would therefore be only one force at work. A force that would manifest itself under a qualitatively distinct phenomenology: “this circular force through a perpetual exchange, in inanimate nature as well as in living nature. In both domains, there is no phenomenon without transformation of force” (Mayer, 1845, In: Assoun, 1981, p. 164), that force remaining constant beyond all its transformations.

If it has therefore been so essential to consider, at such length, the concept in physics of the principle of conservation, it is that, as has so rightly pointed out Paul-Laurent Assoun, “not only do physiology and physics take their inspiration from it, they are also closely involved in its evolution. It is too little to say that physics extends to or is applied to psychophysiology, there is an imbrication of the two” (Assoun, 1981, p. 166). We have been able to observe that the intuition of the conservation of energy had been for Mayer influenced by his medical practice, in connection with his physiological research on ‘body heat.’ It was in effect while meditating on the production of body heat through combustion, that he was able to deduce the principle of conservation. However, after having theorized from a purely physical point of view the conservation of force in ‘inanimate nature,’ he would come back to a physiological application of this energy gain. Thus, “energetism is introduced into psychophysiology not by a simple extension, but as an annex field of verification of one and the same idea” (Assoun, 1981, p. 166) The implication of this is that at no time did Freud feel that he was ‘borrowing’ concepts from physics or physiology, rather he was ‘managing his property,’ in as much as this energetics model was an inherent part of his ‘scientific cradle.’ In this sense, *Project for a Scientific Psychology* (Freud, 1895a) is a paradigmatic example of the importance of this heritage (Assoun, 1981).

While Mayer was the first to establish the principle of conservation of force in physics, and to give an equation for the equivalence between heat and movement, Helmholtz would pursue his project by applying this principle to physiology (Assoun, 1981). Freud would recognize him as his idol – indeed Helmholtz would dominate the German University scene in the 19th century. It was then, essentially through Helmholtz’s work, that Freud would assimilate the principle of conservation of energy, and its applications to physiology. In his memoir of 1847, *On the Conservation of Force*, Helmholtz in the first instance excludes any possibility of ‘perpetual motion’. In this he was going against the generally held view according to which, an inexhaustible and constantly renewed ‘vital force’ would maintain the activities of living organism, and would control the activities of physical and chemical forces (Locqueneux, 2009). The impossibility of perpetual motion had already been justified in mechanics. However, Helmholtz extended it to the whole of nature by applying it to natural forces of a different order to mechanical forces, forces such as heat, electricity, magnetism, light, and chemical reactions: “there does not exist, in all the series of natural actions, a process that would permit the creation of mechanical force without an equal expenditure” (Helmholtz, 1847, In: Locqueneux, 2009, p. 124). Thus it would be impossible to imagine, in nature, a machine that would present perpetual motion – a swing or a pendulum, for example, would eventually stop under the effect of friction and the loss of heat that results (Meulders, 2001). We might be tempted to counter this impossibility with the definition of inertia, posed by Newton as the first law of classical physics, that consist in the tendency of bodies to maintain their speed. However, this universal property only allows for the conceptualization of perpetual motion under abstract conditions, in a closed system, protected from any other force other than the one that had caused the body’s speed. This case is thus utopian, and not observable in nature. It would require the existence of a closed space, in a vacuum, and without friction. Yet, even in this fictional case it would be, as Michel Meulders points out, inappropriate to speak of ‘perpetual motion,’ since at rest bodies are immobile and that only an external force could have put them into motion (Meulders, 2001). There exists then a certain ambiguity, as Pierre Costabel points out, in classical physics’, between its posit of the impossibility of perpetual motion and its definition of inertial movement (Costabel). For Helmholtz then, the impossibility of perpetual motion was correlative to the principle of conservation of force, according to which there could only be creation of movement through a corresponding expenditure of energy. Thus the impossibility of perpetual motion is heir to Leibniz’s dynamics. Leibniz had indeed been the first to profess the impossibility of ‘mechanical’ perpetual motion based on the elementary metaphysical principle that it is impossible to create from nothing, *ex nihilo* (Costabel).

For Helmholtz, all actions of nature must then be brought back, in the final instance, to the opposition of the two forces of repulsion and attraction, as they were formulated by Newton: “thus the problem for the physical sciences consists in bringing all natural phenomena back to invariable forces, attraction and repulsion, whose intensity depends on the distance from the centers of action” (Meulders, 2001, p. 128). On the basis of these two presuppositions (the impossibility of perpetual motion, and reduction of all the actions of nature to the forces of repulsion and attraction), Helmholtz came to establish, in his memoir, the principle of conservation of ‘vital force.’ The problem he was confronted with, and which led him to the definition of this principle, can be summarized by the paradigmatic example of the movement of a swing, which, when it reaches the highest point of its movement, finds itself for a brief moment immobile, before beginning its descent under the influence of gravity. At that point it once again gains speed and goes up in the opposite direction, fighting gravity, before decelerating, and stopping once again under the effect of gravity. The appearance is then that there are two forces involved: one caused by gravity, and the other operating in an opposite direction through the effect of the speed gained by the swing. The first force would be at its maximum when the swing is at the highest point, whilst the other force would reach its paroxysm when the swing goes by, very fast, on the vertical. According to Michel Meulders, “everything seems to the ‘naive’ observer as if these two ‘forces’ to have a mysterious relationship to each other, in which the increase in one would lead to the decrease of the other, and vice versa” (Meulders, 2001, p. 129).

Alongside the ‘live force,’ which here corresponds to the effective movement of the swing, Helmholtz established the necessity to introduce a ‘tension force’ that corresponded to the potentiality of movement to come, when the swing is at rest at its apex. The ‘tension forces’ of material bodies were defined as the product of the forces of attraction or repulsion and the distance that separates those particles. The ‘live forces’ were themselves linked to the movement of these particles (Locqueneux, 2009). The dynamics of Helmholtz was thus entirely reducible to a mechanical description of nature. In this way he referred all the forces present in nature (for which Mayer had compiled a list, as we have seen), examples being electrical, chemical, live and calorific force, back to these mechanical forces that were ‘live force’ and ‘tension force.’ He then referred to the results established by Joules between the loss of mechanical force and the giving off of a quantity of heat (for instance emitted by friction). These results had given a mathematical formula for calculating the rise in temperature (the number of degrees of elevation) in relation to the friction (produced here by the elevation of a weight), which Mayer had been the first to formulate.

By using this quantitative mathematical result for the equivalence between heat and mechanical movement, Helmholtz contended that “the quantity of heat can be increased in an absolute manner by the mechanical forces” (Helmholtz, 1847, In: Locqueneux, 2009, p. 126). From a resolutely mechanistic stand point he then reduced heat to a quantity of movement. He argued that what had up until then been called ‘quantity of heat’ would in fact only be another way of expressing a ‘quantity of live force’ of movement within a substance, as well as the ‘quantity of tension force’ of the internal state of that substance. The first would correspond to free or perceptible heat, whilst the second would correspond the latent heat (Locqueneux, 2009). Heat then, would demonstrate the same distribution between potential forces and their active expression (if we refer to the Aristotelian model), the tension forces and ‘live forces.’ Helmholtz concluded from this that all natural phenomena, whether they applied to organic or inanimate substances, were caused solely by ‘live force’s (*veres vivae*) and tension forces (*Spannkraft*). The concept of force, in a Kantian perspective, thus allowed nature to be rendered intelligible, and to unify the knowledge we have of it (Locqueneux, 2009).

## The Energetics Model

Mayer and Helmholtz made the principle of conservation of force a fundamental principle, one that allowed for the union between physics and physiology. However, the concept of energy does not yet appear in Mayer’s memoir, nor in Helmholtz’s *On the Conservation of Force*, published in 1847. It was only with the introduction of the concept of energy by Thomson, then Rankine, that Helmholtz adopted this new terminology. Helmoltz then rewrote his concepts of ‘live force’ and ‘tension force’ in terms of kinetic energy and potential energy. Terms that would then be taken up by Breuer in his *Studies on Hysteria* (Freud and Breuer, 1895b), and will thus have a notable influence on Freudian theory.

Thomson mentions, for the first time, the word ‘energy’ in 1850. He retained the word ‘force’ to designate Newtonian forces defined by the laws of movement. Energy, would thence designate all the other kinds of force that Mayer had enumerated in his memoir. Thomson considered the existence of two categories of energy, a static energy and a dynamic energy (Locqueneux, 2009). These could also cover the distinction between a latent force and an active force that was already present in Helmholtz’s definition of ‘live forces’ and tension forces. Thus, in his paper *On the universal Tendency in Nature to the Dissipation of Mechanical Energy*. (Thomson, 1852), Thomson states that:

“a load suspended and ready to fall, an electrified body, a quantity of fuel or coal, contain reserves of energy of a static nature, a physical body in motion, an area of space crossed by light or radiant heat waves, a body whose molecules are agitated, contain reserves of energy of a dynamic nature” (Thomson, 1852, In: Locqueneux, 2009, p.127)

It needs to be pointed out that only measurable physical quantities were mentioned here: indeed, the question of the quantification of energy would remain one of the most important objectives for all 19th century German physics. This in opposition to *Naturphilosophie*’s purely qualitative terms for the description of nature.

It was also in this same paper that Thomson expressed for the first time the second principle of thermodynamics. After having regrouped everything that Mayer designated with the term of force (mechanical, chemical, magnetic, calorific…) under the one concept of energy; he turned his attention to the output of real machines, that demonstrated a loss of energy – whereas Sadi Carnot had laid down the grounds for a principle of conservation, working from an abstraction of ideal machines which would experience no reduction in yield. In this way, Thomson concluded that when heat passed by conduction from one body, to another body at a lower temperature, some wastage of mechanical energy would occur. This loss of mechanical energy, through thermal conduction, would have as a consequence, an irreversibility in the processes taking place in thermodynamic machines. This observation would then be generalized to the statement of a continuous degradation of energy in the universe (Locqueneux, 2009). Thus, the irreversible propagation of heat – synonymous in the context of thermodynamic machines with a loss of yield – would become, from 1852 onward, the tendency to the universal degradation of mechanical energy (Prigogine and Stengers, 1979). As Prigogine and Stenger point out: “In this way, Thomson makes the vertiginous leap from the technology of motors, to cosmology […]. Thomson’s new theory […] also makes manifest the consequences of the irreversible propagation of heat in a world where energy is preserved, this world […] can only be at the cost of an irreversible waste, a useless dissipation of a certain quantity of heat. The differences that produce effects are ceaselessly diminishing within nature” (Prigogine and Stengers, 1979, pp. 184–185).

From the 1850s onward, we find the two principles of thermodynamics formulated almost in their definitive forms: the conservation of energy, and the entropy principle. The reformulation by Thomson, of Helmholtz and Mayer’s work on the conservation of force, marked the consecration of the energetics model, and would henceforth dominate the German scientific landscape of the second half of the 19th century. In 1853, Rankine performed some level of synthesis of the Helmoltzian distinction between the ‘live forces’ and the tension forces, and the unifying concept of energy introduced by Thomson, by separating energy into two categories: potential energy (contained in material constructs capable of producing work), and kinetic energy. This separation was applied not only to mechanical force, but also to all kinds of physical phenomena; and covered the Aristotelian concepts of *dynamis* and *energeia*, potency and actuality. In an article written in 1862, Thomson would substitute the term kinetic energy for that of actual energy, and it was this terminology that Helmholtz would use when he went on to adopt the term of energy rather than that of force (Locqueneux, 2009).

Henceforth Helmholtz would favor the concept of energy over that of force, in so far as although the latter remains the ultimate cause of movement, energy as a quantitative concept, measures the capacity of a system to realize, under the impulsion of a force, a certain quantity of work, be it mechanical, caloric, chemical or electrical (Meulders, 2001). To go back to the example of the swing that had illustrated the difference between ‘live force’ and tension force: energy, that is to say the capacity of the swing to perform, under the impulsion of a force, its mechanical pendulum movement, can be considered as the sum of two energies, one kinetic (that of active movement) and the other potential (containing the latent movement). The first would consist of the speed of the given movement, whereas the second would be relative to the position of the swing in space, subject to the forces of gravitation and gravity (Meulders, 2001). Helmholtz held that the sum of the kinetic and potential energies always remained constant: “In all instances of the movement of free material points under the influence of their forces of attraction or repulsion, the intensity of which depends only on distance, the reduction of potential energy is always equal to the increase in live force (kinetic energy). The sum of the live forces and of the potential energy is always constant” (Helmholtz, 1882a,b, In: Meulders, 2001, p. 130).

This integration by Helmholtz of the concept of energy to his previous developments on the principle of conservation of force, and the distinction between ‘live force’ (which will become kinetic energy) and ‘tension force’ (that will subsequently called potential energy), bears witness to the resolutely mechanical character of his references to energetics. Helmholtz, along with Joule or Rankine, used the concept of energy to extend mechanical principles to other non-mechanical domains. This was contrary to Mayer, who saw mechanical phenomena as simply consisting in a particular instance of the phenomena of transformation of energy (Assoun, 1981). It is right to underline that Ostwald, professor at Leipzig since 1887, would specifically oppose this ‘energetic mechanism’ or ‘mitigated energetics,’ raising energetics to the rank of doctrine, assimilable to a quasi-theological *Weltaschaung*, and very close to *Naturphilosophie*. He would propose a new philosophy of science, the fundamental concept of which would be that of energy (Assoun, 1981). His integral energetics formed the basis for an immaterial ontology, and was akin to a radical monism where “nothing seems to be able to occur without energy being a part of it” (Ostwald, 1891, In: Assoun, 1981, p. 170), whereas Mayer had held on to a dualist model, considering matter and force as two distinct entities. Ostwald put himself in opposition to the definition of a potential energy (inherited from the mechanical concept of tension force), that would erase the original and universal reality that is energy in its actuality.

Freud, as we have seen, idolized Helmholtz. Furthermore, he would take as his reference the definition, inspired by Helmholtz, and put forward by Breuer in *Studies on Hysteria* (Freud and Breuer, 1895b), that postulated the existence of a nervous energy, defined as “intracerebral tonic excitation,” the nature of which could be quiescent (that is potential) or kinetic (actual) – although Freud would make some alterations to this definition. Freud’s model then was resolutely that of the mitigated energetics of Helmholtz, in so far as in essence he used a functional energetics applied to the functioning of the psyche, and would regularly use the term ‘work’ to describe the processes of the unconscious (dream work, work of mourning, etc.). His description of the movement between different psychological states, that would entail a mechanical expenditure, would also be a “specific expression of the general rise in disorder that the second principle of thermodynamics formulates” (Assoun, 1981, p. 182). This is why, as Paul-Laurent Assoun points out: “Freud never encounters the temptation, inherent to doctrinal energetics, to exalt energy as a supra-mechanic active principle, and to hypostasize it in support of a world view” (Assoun, 1981, p. 182). Energetics will constitute the basis of all the economic aspect of the metapsychology, but “never will this model of deciphering hypostasize into an energetics doctrine” (Assoun, 1981, p. 182–183).

## From Nervous Energy to Psychic Energy

The introduction in the 1850s of the concept of energy, propelled notably by the influence of Mayer and Helmholtz’s work, appears as the heuristic key for the coming together of physiology and physics. This laid down the foundations, as we have seen, for a resolutely physical epistemological model, which Freud in turn wholeheartedly adheres to. This model positioned itself in a radically autonomous position in relation to the Bernardian revolution in France, which had promoted the singularity and the independence of physiology as a separate science. From then on, Helmholtz, and in his wake Brücke, would give themselves the task of applying the physical principle of the conservation of energy, to organic phenomena. The concept of energy would thus make it possible to simultaneously encompass the concept of force (kinetic, thermic, electrical, magnetic), and phenomena that belonged to living organisms, such as innervation, irritability, and some chemical reactions (Assoun, 1981).

What Helmholtz would aim to achieve in his work *On the Conservation of Force* (Helmholtz, 1847), consisted in applying the concept in physics of conservation of energy, to biology, by making it a postulate for physiological events (Assoun, 1981). The publication of this work marks an essential turning point in accomplishing the unification of the natural sciences, through the application of the principle of conservation of energy. Helmholz can thus be incontrovertibly recognized as the scientist who opened the royal road for an energetics concept of physiology, as well as of psychology. Thus, as Assoun emphasizes, “When in 1883, Freud admits his idolization of the great Berlin master, he is expressing an emotional adherence to a model that confirms his epistemological position. It is, furthermore, with the man who clinched the union of psychology and neurology that he throws in his enthusiastic lot” (Assoun, 1981, p. 158). In this resolutely Helmholtzian filiation, when it comes to a desire to apply the principles of energetics to physiology and anatomy, we can more specifically postulate the probable influence on Freud of Helmholtz’s work on neurons and the speed of propagation of the nervous influx. An area Freud was introduced to when he himself worked on the dissection and observation of nerve cells at the Brücke Institute. Nervous innervation and irritability can thus appear as the energetic manifestations specific to the nervous systems of living organisms.

We have seen that, from an epistemological point of view, Freud did not follow Ostwald’s immaterial ontology, which preached an integral energetics assimilable to a *Weltanschauung*. Rather he positioned himself within a mitigated and a mechanic energetics, in a Helmhotzian filiation. Nevertheless, it is right to recognize that Ostwald himself had naturally come to question the possibility for applying the notion of energy to psychological phenomena, to the extent that, in his manifest on *Energy*, he considered the phenomenon of life as a “constant manifestation of energy” (Ostwald, 1891, In: Assoun, 1981, p. 170). Faithful to his pan-energetics view of nature, Ostwald was led to state that “psychological phenomena can be construed as energetic phenomena, and interpreted as such just as well as any other phenomena” (Ostwald, 1891, In: Assoun, 1981, p. 172). Ostwald then, argued for the existence of a nervous energy, and described a process during psychological activity that gives rise to a consumption of energy. In so far as he introduced the concept of ‘psychic energy,’ he can be said to open up the way for Breuer and Freud’s studies on the energy of the nervous system, or “intracerebral tonic excitation” (Freud and Breuer, 1895b) for which Freud would give the term ‘quantity’ in his *Project for a Scientific Psychology* (Freud, 1895a). Ostwald would thus have been the first to see psychic phenomena as being “phenomena of nervous energy.” Going on to define them as being, like all energy, a “measurable quantity that obeys the law of conservation and that of transformation” (Ostwald, 1891, In: Assoun, 1981, p. 172), that could manifest themselves under diverse forms. This need for quantification and measurement of psychic energy will continue throughout the work of Freud. Freud would, however, renounce giving it an absolute value, and accommodate himself to attributing to it a measure that was only relative. The principle of conservation of energy was thus applied by Ostwald to psychological phenomena in so far as, according to him “no psychological operation takes place without a corresponding consumption of energy” (Ostwald, 1891, In: Assoun, 1981, p. 172). Freud would reapply this introduction of the principle of conservation of energy in the field of psychology, but without following the metaphysical consequences that Ostwald upheld (that is to say the circumventing of the “religious problem of the soul and the body” through the concept of psychic energy).

It is then, these models for the implementation of energetics in physiology, and *a fortiori* psychology, that Freud, in the wake of Helmholtz, would pursue. This was not, however, without some influence from Ostwald; although he in no way adhered to Ostwald’s ontological monism, and would retain all through his work a mechanical energetics close to that of Helmholtz. This physicalist paradigm would be enriched in Freud’s thinking by his training in anatomy at the Brücke Institute, where he devoted himself to the meticulous study of nerve cells. As Ernest Jones recounts, Freud, then a medical student, was seduced by the psycho-physiological theories held at the Brücke Institute – Brücke supported Helmholtz’s school, and also played an important role at his side in the Berlin Physical Society (Jones, 1953). Du Bois-Reymond reports that Helmholtz and Brücke had “pledged a solemn oath to put into effect this truth: “No other forces than the common physical-chemical ones are active within the organism.” ” (Jones, 1953, p.40). The training that Freud received at the Brücke Institute was, then, dominated by the application of the principle of conservation of energy to organisms: “Organisms differ from dead material entities in action – machines – in possessing the faculty of assimilation, but they are all phenomena of the physical world; systems of atoms, moved by forces, according to the principle of the conservation of energy discovered by Robert Mayer in 1842, neglected for 20 years, and then popularized by Helmholtz. The sum of forces (motive forces and potential forces) remains constant in every isolated system. The real causes are symbolized in science by the word ‘force’.” (Jones, 1953, p.41).

Here is then, summed up by Jones, the message of the German school of physiology at the heart of which Freud will be immersed during his years of training. The works of Brücke devoted to transformation and to the effects of physical forces in the living organism, will have a lasting influence on the dynamic view of metapsychology; and Freud, up until 1926, would argue that within the psychical apparatus “The Forces assist or inhibit one another, combine with one another, enter into compromises with one another, etc.” (Jones, 1953, p. 42).

## Conclusion

The roots of the Freudian energetics model, heavily influenced by the discovery of the principle of conservation of force in thermodynamics, prompt us to understand to origins of the theoretical model that he develops starting with in *Project for a Scientific Psychology* (Freud, 1895a), not as a primarily biological model, but on the contrary as a paradigm radically grounded in physics. Freud’s developments on the principle of inertia, then on the pleasure principle, and later, on the death drive, should then, be re-situated within the scientific project of the Helmholtz school, a project to subordinate physiology, and subsequently psychology, to an ideal of physics. Not disregarding the fact that, in the 19th century German and Austrian scientific context, biology had not yet acquired its independence from the ideal of physics; the model put forward in *Project for a Scientific Psychology* (1895a) should therefore not be too swiftly described as exclusively biological. The physiology training that Freud received at the Brücke Institute is in no way comparable to the Bernardian physiology. Bernard had gone in a different direction introducing a new position. While rejecting vitalism, Claude Bernard admitted the existence of a singularity of the ‘vital force,’ allowing for an autonomy of experimental physiology as an independent science in relation to the science of physics.

It is within a biology that is firmly subordinated to the *Berliner Physikalische Gesellschaft’s* ideal of physics, dominated by the influence of Helmholtz, that the energetics model of the *Project for a Scientific Psychology* (Freud, 1895a) is rooted. These considerations could open up a field of enquiry that would benefit from further investigation: If Freud, notably in the wake of the publication of The Interpretation *of Dreams* (Freud, 1900a), renounces - at least temporarily – grounding his theorizing on a biological model, is this move also accompanied by a renouncement of the physicalist epistemological foundations of that model? This epistemological turning point, already announced in the letter to Fliess dated 6th December 1896 (Freud, 1950 [1892–1899]), seems to represent a turning point in the move from a “neuronal apparatus” model in the *Project for a Scientific Psychology* (Freud, 1895a), to the abandonment of this project for basing mental processes on a precise description of the nervous system. In *The Interpretation of Dreams* (Freud, 1900a) Freud will have indeed abandoned the vocabulary of physiology, no longer referring to the structure and anatomy of neurons, and will henceforth refer exclusively to a “psychical” apparatus. In chapter VII, he formulates this renouncement of an anatomically localized model thus:

“I shall entirely disregard the fact that the mental apparatus with which we are here concerned is also known to us in the form of an anatomical preparation, and I shall carefully avoid the temptation to determine psychical locality in any anatomical fashion.” (Freud, 1900a, p. 536)

This is only a temporary renouncement, in so much as that the hope for a physiological model is deferred to such a time as progress in biology will make it possible to precisely base psychical processes on a physical substrate. Nevertheless, this research had henceforth become secondary for Freud, and no longer constitutes the primary aim of his theorization: “We may, I think, dismiss the possibility of giving the phrase an anatomical interpretation...” (Freud, 1900a, pp. 48–49)

Thus, although reference to anatomy is not totally absent from Freud’s thinking after 1899, it remains a fact that this search for an anatomical location is sidelined from a topical point of view from the metapsychology. In many ways it is no longer necessary to metapsychology, which can do without it. One question does remain: Is this putting aside (however, temporary it may be) of all references to an anatomical location for the psychical processes accompanied by a renunciation of the physics model? It was this physical model that had enabled him, progressively, to draw out, on the basis of the idea of nervous energy, the concept of psychical energy, that will subsequently become libido. We could be tempted to argue that, despite abandoning a biological reference in the construction of Freudian metapsychology, the influence of a physics epistemological model seems to remain. However, this question is no the object of this research, and will be the focus of work to come.

These historical elements can, then, prompt us to reconsider the major metapsychological concepts through the prism of the energetics model. In particular the important principles of thermodynamics as they were understood when they came to light at the end of the 19th century. A further in-depth look at the Freudian formulations of the principle of inertia, the pleasure principle and even the death drive, will be enriched by an analysis that seeks to bring to light the links between these concepts and the influence of the physics model, more particularly the model of thermodynamics at the roots of Freudian thinking.

## Author Contributions

JT is the main contributor of this paper as part of her Ph.D. thesis. FA and PM as supervisors, contributed to the conception and development of the research, and they revised critically the manuscript for intellectual content.

## Conflict of Interest Statement

The authors declare that the research was conducted in the absence of any commercial or financial relationships that could be construed as a potential conflict of interest.
